# Angioedema

**DOI:** 10.1186/s13223-024-00934-3

**Published:** 2024-12-09

**Authors:** Gina Lacuesta, Stephen D. Betschel, Ellie Tsai, Harold Kim

**Affiliations:** 1 Halifax Allergy and Asthma Associates, Department of Medicine, Dalhousie University, Halifax, NS Canada; 2 Division of Allergy and Immunology, Department of Medicine, St. Michael’s Hospital, University of Toronto, Toronto, ON Canada; 3 Division of Allergy and Immunology, Department of Medicine, Queen’s University, Kingston, ON Canada; 4 Division of Allergy and Immunology, Department of Medicine, Western University, London, ON Canada; 5 Division of Allergy and Immunology, Department of Medicine, McMaster University, Hamilton, ON Canada

**Keywords:** Angioedema, Hereditary angioedema, Acquired angioedema

## Abstract

Angioedema can occur in the absence of urticaria and can be broadly divided into three main categories: mast cell-mediated (e.g., histamine), non-mast-cell-mediated (bradykinin-induced) and idiopathic angioedema. Non-mast-cell-mediated angioedema is largely driven by bradykinin. Bradykinin-induced angioedema can be hereditary, acquired or drug-induced, such as with angiotensin-converting enzyme (ACE) inhibitors. Although bradykinin-mediated angioedema can be self-limited, it can cause significant morbidity and laryngeal involvement may lead to fatal asphyxiation. The mainstays of management for angioedema are: (1) to avoid specific triggers (if possible and where known) and (2) treatment with medication (if indicated). For hereditary angioedema (HAE), there are specifically licensed treatments that can be used for the management of attacks, or for prophylaxis in order to prevent attacks. In this article, the authors will review the causes, diagnosis and management of angioedema.

## Key take-home messages

- Angioedema can occur in the absence of urticaria, with ACE inhibitor-induced and idiopathic/spontaneous angioedema being the most common causes.
- ACE inhibitors should be discontinued in any individual who presents with angioedema without urticaria as this condition is associated with life-threatening upper airway angioedema.
- Idiopathic/spontaneous angioedema responds well to prophylactic antihistamines (which may be increased to fourfold the standard dosing); however, more advanced treatment, such as omalizumab, may be needed. In some acute situations, oral corticosteroids may be required.
- HAE and AAE are rare disorders also characterized by angioedema in the absence of urticaria; they result from a deficiency or dysfunction of the C1-INH (a plasma protease inhibitor that regulates several proinflammatory pathways) and can be associated with life-threatening upper airway swelling.
- The diagnosis of HAE and AAE should include the assessment of C4, C1q, and C1-INH function and antigenic levels.
- The management of HAE and AAE involves an approach to acute treatment, short-term and long-term prophylaxis that is evidence-based and follows current guideline recommendations.
- All patients suspected of having HAE or AAE should be referred to, and managed by, a specialist with expertise in these conditions.

## Introduction

**Fig. 1:**
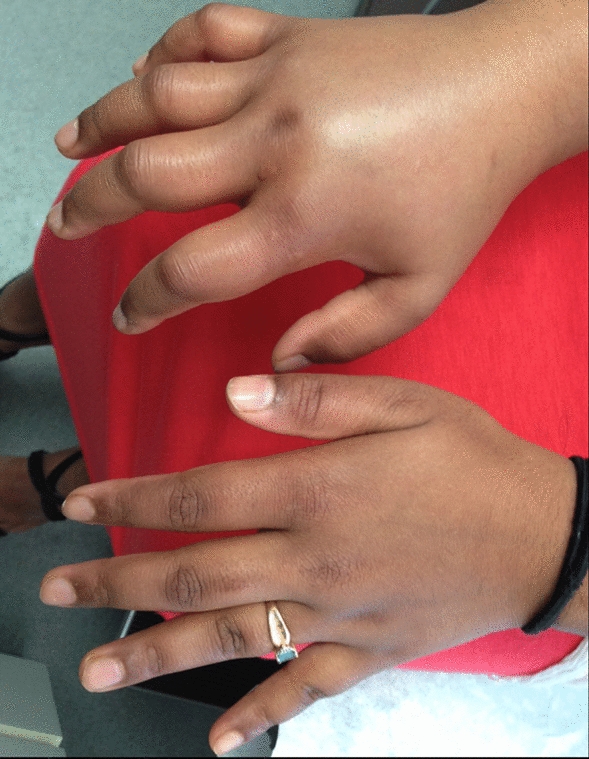
Hereditary angioedema (HAE)

**Fig. 2:**
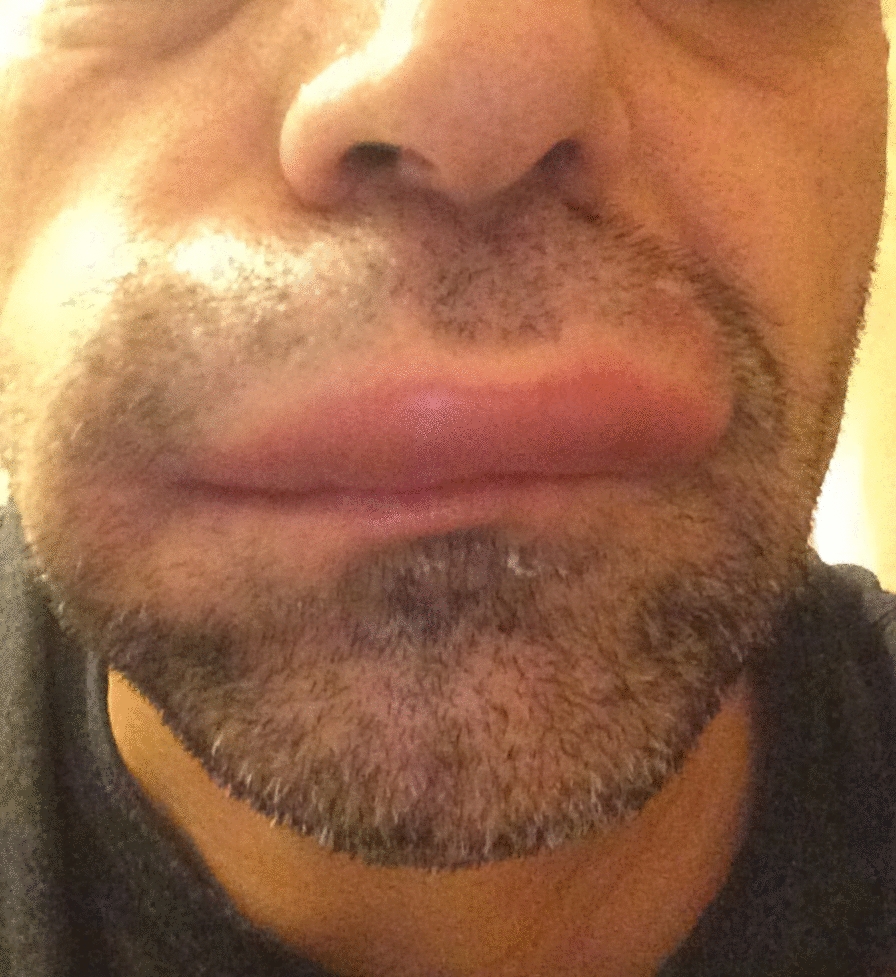
Mast cell-mediated angioedema

Angioedema in the absence of urticaria (see Figs. 1 and 2) is much less common than urticaria, either with or without angioedema. Its presence should alert the physician to alternative diagnoses, which could comprise life-threatening conditions that are refractory to the usual first-line treatments in the emergency room (i.e., antihistamines, corticosteroids, epinephrine), including hereditary angioedema (HAE), acquired angioedema (AAE), or angioedema associated with angiotensin-converting enzyme (ACE) inhibitors.

**Fig. 3:**
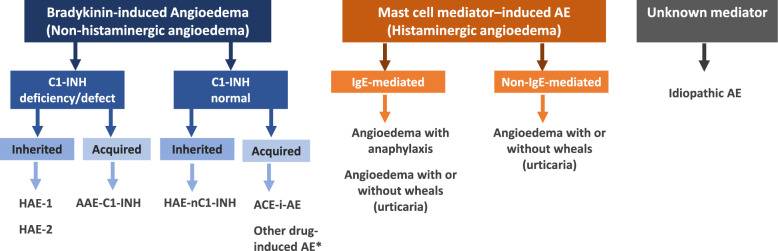
Classification of angioedema [1]. *Other drugs like gliptins, neprilysin inhibitors or tissue plasminogen activators are thought to potentially induce bradykinin-mediated AE. AE: angioedema; HAE-1: hereditary angioedema due to C1-inhibitor deficiency; HAE-2: hereditary angioedema due to C1-inhibitor dysfunction; AAE-C1-INH: acquired angioedema due to C1-inhibitor deficiency; HAE-nC1-INH: hereditary angioedema with normal C1-Inhibitor levels, ACEI-AE angiotensin-converting enzyme inhibitor-induced angioedema; IgE, immunoglobulin E Adapted from: Maurer 2022 [1].

Episodes of angioedema result from a release of vasoactive mediators that increase vascular permeability in the skin and submucosa, allowing for the vascular leakage of plasma and resultant edema; the majority of these attacks can be attributable to either mast cell-mediated (e.g., histamine) or bradykinin-mediated mechanisms. Angioedema can be further classified as: mast cell-mediated (i.e., through immunoglobulin E [IgE] -independent or dependent mechanisms), idiopathic, hereditary (HAE type I [HAE-1], HAE type II [HAE-2] and HAE with normal C1 inhibitor [HAE-nC1-INH]), acquired (C1-inhibitor [C1-INH] deficiency from a secondary cause) and drug-induced (e.g., ACE inhibitors) (see Fig. 3 for classification of angioedema) [1]. This article will briefly discuss mast cell-mediated angioedema, but will focus on the classification, diagnosis and management of bradykinin-mediated angioedema.

### Mast cell–mediated angioedema

The approach to mast cell-mediated (e.g., histamine) angioedema is similar to that of acute urticaria (see *Urticaria* article in this supplement) and includes avoidance measures, antihistamines (preferably second-generation antihistamines) and, if needed, corticosteroids. It is important to note that patients with allergic angioedema may not always have urticaria accompanying their angioedema. Approximately 40% (range 33–67%) of patients with urticaria develop angioedema [2]. In a significant number of patients, no identifiable cause is found for recurrent episodes of angioedema without urticaria; this is deemed as spontaneous angioedema once alternate identifiable causes have been excluded. Spontaneous cases may occur in antibody-dependent and independent mechanisms, and typically respond to antihistamines (up to a four-fold increase in the standard dose of antihistamine may be required in those who are unresponsive to the standard dose) [3], corticosteroids and more advanced treatment with omalizumab (a monoclonal anti-immunoglobulin E [IgE] antibody) [4]. Diagnosis is often made after ruling out other etiologies and by assessing a patient’s response to mast cell-mediated treatments. Clinical features differentiating mast cell-mediated and bradykinin-mediated angioedema are summarized in Table 1 [5, 6].

**Table 1:** Clinical features differentiating mast cell-mediated and bradykinin-mediated angioedema [5, 6]

Characteristics	Mast cell-mediated angioedema	Bradykinin-mediated angioedema
Speed of onset	Minutes to hours	24–36 h
Attack duration (without treatment)	Several hours	3–5 days
Predominant attack location	Common locations include the face (eyelids, lips) and extremities	Extremities, face, upper airways, genitourinary tract, GI tract/bowel wall
Urticaria/pruritus	Common	Rare
Triggers	Allergies (e.g., insect stings), stress, medications (e.g., NSAIDS/ASA), often spontaneous	Trauma, infections, stress, hormonal changes (e.g., estrogen, pregnancy), medications (i.e., ACE-i), can also be spontaneous
Response to antihistamines, corticosteroids and epinephrine	Yes	No

*HAE* hereditary angioedema; *GI* gastrointestinal; *ACE-I* angiotensin-converting enzyme inhibitor; *NSAIDs* non-steroidal anti-inflammatory drugs; *ASA* acetylsalicylic acid

### Bradykinin-mediated angioedema

In the case of HAE, AAE or ACE inhibitor-induced angioedema, the vasodilatory peptide, bradykinin, plays a key role in endothelial cell activation, with resultant tissue edema. Bradykinin is released from many cell types, and mechanisms that contribute to either its overproduction or, in the case of ACE inhibitors, its degradation, result in angioedema. This may occur through several mechanisms, namely the complement, coagulation and contact pathways, which are essential in the regulation of bradykinin.

#### Classification and etiology

HAE is a rare autosomal dominant genetic disorder resulting from an inherited deficiency or dysfunction of the C1 inhibitor (C1-INH) which is a plasma protease inhibitor that regulates several proinflammatory pathways. There are three types of HAE (see Table 2) [7]. The two main types of HAE are: type I (HAE-1), which is characterized by low C1-INH levels and function (85% of cases), and type 2 (HAE-2), which is associated with normal C1-INH levels but low function (15% of cases). The third type is HAE with normal C1-INH level and function (HAE-nC1-INH). This type comprises a group of rare diseases thought to be mainly bradykinin mediated. There are 6 recognized underlying gene mutations for HAE-nC1-INH [1]; the most common is the female predominant, gain of function mutation in factor X11(FXII). However, there are many patients with HAE-nC1-INH where no gene mutation has been found.

**Table 2:** Comparison of HAE and AAE [7]

	Family history	Complement levels/laboratory findings			
		C4	C1-INH antigen	C1-INH function	C1q^†^
HAE-1 HAE-2	Yes*	↓ ↓	↓ Normal or ↑	↓ ↓	Normal
HAE-nC1-INH	Yes	Normal	Normal	Normal	Normal
AAE	No	↓	Normal or ↓	↓	Low

*In approximately 25% of patients, no family history is identified; the disorder results from spontaneous mutation of the C1 inhibitor gene

^†^C1q level testing may be difficult to obtain in Canada

Adapted from: Betschel et al. 2019 [7]

AAE is a rare C1-INH deficiency syndrome most commonly associated with B-cell lymphoproliferative diseases (type 1 AAE) or autoimmune conditions [8]. It may also be related to the presence of an autoantibody directed against the C1-INH molecule (type II AAE).

Clinically, HAE and AAE are similar, and are characterized by recurrent episodes of angioedema, without urticaria or pruritus, which most often affect the skin or mucosal tissues of the gastrointestinal and upper respiratory tracts. Peripheral swelling is also common. Although these can be self-limited, the angioedema can cause significant morbidity and decreased quality of life (QoL) [1, 7]. Abdominal pain and other abdominal symptoms such as nausea, vomiting and diarrhea are common in patients with HAE, and these symptoms often prompt patients to seek emergency medical attention. Laryngeal involvement can rapidly lead to fatal asphyxiation if left untreated.

Age of onset and the presence of a familial history are distinguishing features between HAE and AAE (see Table 2). HAE usually presents in late childhood or adolescence in otherwise healthy subjects, and a familial history is present in approximately 75% of cases (with the remaining 25% resulting from spontaneous mutation of the C1-INH gene) [1, 7]. In contrast, AAE is not associated with a family history, and usually develops in older patients (fourth decade of life and older) with an underlying lymphoproliferative or autoimmune disease [8].

As mentioned earlier, excess production of the potent vasodilatory peptide, bradykinin (which is regulated by the C1-INH), plays a key role in the pathogenesis of HAE-1 and -2 and AAE [7]. It is important to note that histamine and other mast-cell mediators that are typical of urticaria and associated angioedema are not directly involved in HAE and AAE, which explains patients’ lack of response to antihistamines and corticosteroids, and distinguishes these forms of isolated angioedema from that associated with urticaria.

ACE inhibitor-induced angioedema is one of the most common causes for emergency treatment of angioedema; it occurs in approximately 0.1–6% of individuals using ACE inhibitors and tends to occur more commonly in ACE inhibitor users who are female, smokers, or of African-American descent [9]. Like HAE and AAE, ACE inhibitor–induced angioedema is bradykinin-mediated. Most cases of angioedema occur in the first week after starting ACE inhibitor therapy. However, up to one-third of cases occur months to years after initiating the medication [10]. ACE inhibitor-induced angioedema can be life-threatening when it involves the upper airway. Therefore, ACE inhibitors should be discontinued in all individuals with angioedema without urticaria and are absolutely contraindicated in patients with either HAE or AAE. Episodes of angioedema may occur up to 1 month (or sometimes more) after discontinuing the ACE inhibitor. Angiotensin receptor blockers (ARBs) typically do not promote bradykinin accumulation and, therefore, are not contraindicated in these patients and can be used if indicated.

#### Diagnosis

The diagnosis of HAE and AAE is based on a suggestive clinical history, and there is significant overlap in their clinical presentation. The angioedema may present in the face, extremities, abdomen and other organ systems, with the concern of laryngeal edema and asphyxiation. The most common presentation is that of recurrent angioedema in the absence of associated urticaria resulting in impairment in QoL with discomfort, immobility and disfigurement, and the inability to attend work or school [11, 12].

Abdominal attacks occur in up to 93% of patients with HAE [13]. These attacks can present with mild to severe spasmodic pain, and may be associated with gastrointestinal upset and even intestinal obstruction as well as ascites; hypovolemic shock may result from the extravasation of fluids. The attacks can often be confused with appendicitis or cholecystitis, resulting in unnecessary surgical interventions, and even psychiatric referrals.

Laryngeal attacks with respiratory impairment and the risk of asphyxiation are the most feared complications of these attacks, as these patients may require intubation and even tracheotomies. It is estimated that up to 50% of HAE patients will experience at least 1 laryngeal episode within their lifetime [14].

Complement studies that should be ordered for patients with suspected HAE and AAE include levels of C4 (the natural substrate for C1), C1q, C1-INH antigen, and function of C1-INH (Table 2) [7]. Ideally, these studies should be performed when the patient is not receiving treatment since the use of therapeutic interventions for AAE or HAE can alter laboratory results. Also, the sensitivity of these tests increases when performed during an active angioedema attack [1]. If clinical suspicion of HAE is high based on the initial testing, these tests should be repeated to help confirm the diagnosis. All offspring and first-degree relatives of patients with HAE should be considered to have HAE until proven otherwise and should be screened [7]. Patients with AAE should be evaluated for an underlying B-cell lymphoproliferative disorder or other hematologic or autoimmune condition at the time of diagnosis [8].

In HAE-1, C1-INH antigenic and functional levels are low (< 50% of the lower limit of normal) (Table 2) [7]. In HAE-2, C1-INH functional levels are low, but antigenic levels are normal or elevated. In HAE-nC1-INH, C4, C1-INH level and function, and C1q are all normal. If HAE-nC1-INH is suspected, patients should be sent for genetic testing (if possible) to look for the known gene variants associated with this disease. It is recognized that this genetic testing is not commercially available in Canada. However, patients can be referred to an HAE specialist who may be able to access genetic testing in specialized research centres.

Criteria for diagnosis of HAE-nC1-INH include: (1) a history of recurrent angioedema in the absence of associated hives or use of medication known to cause angioedema; (2) documented normal or near normal C4, C1-INH and C1 -INH function, and (3) either a genetic variant associated with the disease or a family history of angioedema and documented lack of efficacy of chronic high-dose antihistamine therapy [7]. Healthcare providers should also have a strong index of suspicion for HAE-nC1-INH in patients who fit the above criteria and have failed corticosteroids and/or omalizumab (anti-IgE therapy).

In most patients with AAE, C4, C1q, and C1-INH function levels are low (< 50% of the lower limit of normal), and C1-INH antigenic levels are low or normal.

ACE inhibitor-induced angioedema should be suspected in any patient who is on an ACE inhibitor and develops angioedema without urticaria. Although complement studies are normal in ACE inhibitor-induced angioedema, C1-INH levels should be measured in these patients as the initiation of an ACE inhibitor could unmask HAE or AAE.

All patients suspected of having HAE or AAE should be referred to a specialist with expertise in these conditions. The Canadian Hereditary Angioedema Network provides contact information for angioedema specialists across Canada [15].

#### Treatment of HAE-1/-2 and AAE

Most evidence for managing bradykinin-mediated angioedema is based on clinical trials in HAE. The management of HAE can be divided into the following approaches: treatment of attacks, short-term prophylaxis (STP), and long-term prophylaxis (LTP). The aim of treatment of attacks, also referred to as ‘on demand therapy’, is to minimize their severity, including potentially fatal upper airway angioedema and associated impairment of QoL. STP refers to treatment meant to minimize the risk of attacks when avoidance of potential and known triggers is not possible. LTP refers to ongoing treatment of HAE aimed at minimizing the overall number, frequency and/or severity of attacks and improving QoL [7]. Table 3 lists the recommended treatments for HAE supported by high levels of evidence [1, 7, 16].

**Table 3:** Treatments for HAE available in Canada that are supported by a high level of evidence [7, 16]

HAE-specific treatment	Product name	Mechanism of action	Approved indications in Canada	Dose and route of administration	Age indications
Plasma-derived C1-INH†	Berinert	Replaces C1-INH	Acute treatment	20 U/kg intravenous as needed	Pediatric and adult
	Cinryze	Replaces C1-INH	Long-term prophylaxis	1000 U intravenous q 3–4 days	≥ 12 years
	Haegarda	Replaces C1-INH	Long-term prophylaxis	60 U/kg body weight twice weekly subcutaneously (every 3–4 days)	≥ 12 years
Icatibant	Firazyr	Synthetic selective and specific antagonist of bradykinin 2 receptor	Acute treatment	30 mg subcutaneous injection as needed; dose-adjusted for adolescents < 65 kg and children ≥ 2 years*	≥ 2 years
Lanadelumab	Takhzyro	Fully human monoclonal antibody that binds plasma kallikrein and inhibits its proteolytic activity	Long-term prophylaxis	300 mg subcutaneous injection every 2 weeks A dosing interval of 300 mg every 4 weeks may be considered if the patient is well-controlled (e.g., attack free) for more than 6 months	≥ 12 years
Berotralstat	Orladeyo	Oral, selective inhibitor of plasma kallikrein that blocks enzymatic activity of plasma kallikrein in releasing bradykinin	Long-term prophylaxis	150 mg daily taken orally with food	≥ 12 years

^†^Treatment of choice during pregnancy, delivery and breastfeeding

*12 kg to 25 kg: 10 mg (1.0 ml); 26 kg to 40 kg: 15 mg (1.5 ml); 41 kg to 50 kg: 20 mg (2.0 ml); 51 kg to 65 kg: 25 mg (2.5 ml); > 65 kg: 30 mg (3.0 ml)

Adapted from: Betschel S, et al. Allergy Asthma Clin Immunol. 2019 [7]. Berotralstat product monograph 2022 [16]

##### Acute treatment/treatment of attacks

First-line therapies for the treatment of attacks of HAE and AAE include plasma-derived C1-INH replacement therapy (pdC1-INH), icatibant and ecallantide [7]. The goal of acute treatment is to treat early to reduce the duration and severity of an attack, minimize the impact of an attack on the functional ability of the patient, and reduce morbidity and potential mortality. All patients should have access to an acute therapy that facilitates early treatment and that is best suited to their individual needs. Ideally, this would entail self-treatment. Since these patients may also present to healthcare providers who may not be familiar with their condition, it is advisable that patients carry a wallet card or a similar tool which outlines their condition and their specific treatments.

Treatment with pdC1-INH replaces the deficient protein in patients with HAE-1 and HAE-2. Berinert is the only approved pdC1-INH replacement product in Canada to treat acute attacks. It is administered as an intravenous rapid push at a dose of 20 units/kg. Ruconest, a recombinant form of C1-INH is available in other countries, but not approved for use in Canada. Although pdC1-INH replacement is used to treat AAE, some patients may become non-responsive to this treatment over time. In these patients, the use of ecallantide and icatibant (described below) could be considered.

Icatibant is a bradykinin receptor blocker which is approved in Canada for the treatment of acute attacks of HAE-1 and -2. The usual recommended dose for adults is 30 mg subcutaneously. It is also indicated for ages as low as 2 years, with weight-based dosing for children. Although evidence suggests that icatibant is also effective for the treatment of ACE inhibitor-induced angioedema [17], this is not an approved indication. The most common side effects of icatibant are mild and transient injection-site reactions. Other less common side effects include nausea, gastrointestinal upset, asthenia, dizziness, and headache [7].

Ecallantide, which has not been approved in Canada, is an inhibitor of plasma kallikrein (the enzyme that releases bradykinin, the primary mediator of angioedema). Although the side effects of ecallantide are generally mild (i.e., injection-site reactions, headache, nausea, fatigue, diarrhea), this therapy has been associated with rare instances of allergic reactions and anaphylaxis. Therefore, it should only be administered by a clinician in a medical setting equipped to manage anaphylaxis and severe angioedema [7].

##### Short-term prophylactic treatment (STP)

STP refers to the practice of treating patients to reduce the risk of associated and consequent morbidity and mortality during a period when there may be an increased risk of having an angioedema attack [7]. Triggers for attacks include physical trauma, such as that which may occur during medical and dental procedures. Attacks can occur anywhere from hours to several days after a procedure. Upper airway manipulation (e.g., dental surgery and intubation) is considered particularly high risk due to its association with upper airway swelling. It is also suspected that other causes, such as emotional stressors, can precipitate attacks. Individual patients may also be aware of specific triggers known to trigger their attacks.

STP should be considered prior to exposure to known patient-specific triggers and for any medical, surgical and dental procedures. HAE specific treatment should be available during and after any procedure. Pre-procedural prophylaxis with pd-C1 inhibitor is recommended as first-line treatment at a dose of 20U/kg IV within an hour of the procedure [7].

As a second-line alternative, attenuated androgens may be considered for STP when surgery-related risks are considered low and other HAE-specific acute treatments are not immediately available. If androgens are selected for STP, danazol (2.5–10 mg/kg/day, maximum 600 mg/day) can be considered and should be initiated 5 days before the anticipated procedure or trigger and continued 2–3 days after the anticipated trigger [7].

##### Long-term prophylactic treatment (LTP)

The goal of LTP is to reduce the frequency and/or severity of attacks and minimize the impact of the HAE or AAE on patient QoL, thereby enabling patients to lead normal lives. There are no specific criteria for determining when LTP should be initiated; the decision depends on multiple factors, including the frequency of attacks, the severity of previous attacks, how readily patients can access emergency treatment, their ability to administer on-demand therapy, and the impact on QoL [7]. Since patients on LTP can still have acute attacks, they must have access to acute treatment despite being on prophylaxis.

Factors triggering acute attacks of AAE and HAE vary but can include: dental and surgical procedures, stress/anxiety, infection, menstruation, and the use of estrogen-containing medications (e.g., hormone replacement therapy and contraceptives) and ACE inhibitors. Whenever possible, these triggers should be avoided. For AAE, treating underlying related conditions (i.e., hematologic malignancies or autoimmune disorders) will help decrease angioedema attacks [8].

The most recent international guidelines recommend plasma-derived C1-INH (pdC1-INH), lanadelumab and berotralstat as first-line therapies for LTP in HAE-1 and -2 [1]. All three therapeutic agents are licensed and indicated in Canada for LTP (Table 3). The recommended dose of subcutaneous pdC1-INH is 60 units/kg every 3 to 4 days. Lanadelumab, a fully human plasma kallikrein inhibitor, is administered at 300 mg every 2 weeks. If patients remain asymptomatic, the dose frequency can be decreased to 300 mg every 4 weeks. Side effects of lanadelumab are minimal and typically involve mild, local injection-site reactions. Berotralstat, an oral kallikrein inhibitor, is administered at a dose of 150 mg daily. The side effects of berotralstat are typically mild and may include abdominal pain, vomiting, and diarrhea that occur after initiation of treatment; these side effects tend to become less frequent or resolve over time. The selection of which agent to use for LTP is dependent on patient preference and tolerability, and the decision should be made between the HAE specialist and the patient.

Attenuated androgens (e.g., danazol) increase C4 and C1-INH levels and are considered second-line therapy for LTP in HAE [7]. Although generally well-tolerated by most patients, the adverse effects of long-term androgen administration may include: virilization, abnormalities in serum transaminases, menstrual irregularities, hair growth, decreased libido, weight gain, vasomotor symptoms, lipid abnormalities, hepatocellular adenoma and carcinoma and depression/emotional lability. Therefore, the lowest effective dose should be utilized (the maximum long-term recommended dose for danazol is 200 mg daily), and the patient’s complete blood count (CBC), liver enzymes and lipid profile should be monitored regularly (e.g., every 6 months), along with annual abdominal ultrasound, while on therapy. Contraindications to androgen therapy include: pregnancy, lactation, cancer, hepatitis, and childhood.

There is less evidence to support the use of the antifibrinolytic agent, tranexamic acid, for the prophylactic treatment of HAE and AAE [1, 7]. Tranexamic acid is well-tolerated; however, because of the lack of good efficacy data, it is generally only considered when first-line prophylactic treatments are not available and androgens are contraindicated [1]. The most common side effect is dyspepsia, which can be reduced by taking the drug with food.

#### Treatment for HAE-nC1-INH

Due to the similar clinical presentations of HAE-1/-2 and HAE-nC1-INH, and given that all three types are thought to be bradykinin-mediated, treatment of acute episodes, and STP and LTP in HAE-nC1-INH are the same as for HAE-1/-2 [7]. There are case series showing that the treatments for HAE-1/-2 also work in HAE-nC1-INH [18]. It is also important to avoid angioedema triggers in HAE-nC1-INH, such as estrogen-containing oral contraceptives or estrogen replacement therapy, dipeptidyl peptidase-4 (DPP-4) inhibitors, neprilysin inhibitors and ACE inhibitors.

## Conclusions

Angioedema can occur in the absence of urticaria. The more common causes are ACE inhibitor-induced angioedema and idiopathic/spontaneous angioedema. Rare but life-threatening causes are HAE or AAE. The work-up and management of HAE and AAE vary considerably from that of angioedema associated with urticaria. Although the angioedema associated with these disorders can be self-limited, it can cause significant morbidity, and laryngeal involvement can lead to fatal asphyxiation. Patients with these disorders demonstrate characteristic abnormalities in certain complement levels and, therefore, diagnostic testing of patients with suspected HAE or AAE should include assessment of C4 and C1q levels, and C1-INH function and antigenic levels. HAE should be considered in patients with an early age of onset and a family history of the disorder. In patients with AAE, there is no family history and age of onset is usually later. HAE-nC1-INH should be considered if the C4, C1-INH level and function are all normal, but there is a family history of recurrent angioedema with poor response to high-dose antihistamines and/or corticosteroids. All patients suspected of having HAE or AAE should be referred to, and managed by, a specialist with expertise in these conditions.

Patients with HAE and AAE must have access to effective acute treatment, and measures should be taken to minimize the time required for the administration of acute therapy. Ideally, self-treatment should be available. STP must be considered for all patients with HAE or AAE during times when the risk of angioedema is increased. The decision to start LTP should be based on shared decision-making between the patient and the angioedema specialist after consideration of several factors, including the frequency of attacks, the severity of previous attacks, how readily patients can access emergency treatment, their ability to administer on-demand therapy, and impact on QoL.

## Data Availability

Not applicable.

## Abbreviations

ACE: Angiotensin-converting enzyme
HAE: Hereditary angioedema
HAE-1: Hereditary angioedema type I
HAE-2: Hereditary angioedema type II
AAE: Acquired angioedema
QoL: Quality of life
NSAIDs: Non-steroidal anti-inflammatory drugs
CBC: Complete blood count
HAE-1: Hereditary angioedema type 1
HAE-2: Hereditary angioedema type 2
HAE-nC1-INH: Hereditary angioedema with normal C1 inhibitor
C1-INH: C1-inhibitor
STP: Short-term prophylaxis
LTP: Long-term prophylaxis
